# Dimeric human sulfotransferase 1B1 displays cofactor-dependent subunit communication

**DOI:** 10.1002/prp2.147

**Published:** 2015-05-08

**Authors:** Zachary E Tibbs, Charles N Falany

**Affiliations:** The Department of Pharmacology and Toxicology, The University of Alabama at BirminghamBirmingham, Alabama, 35294-0019

**Keywords:** 3′-Phosphoadenosine 5′-phosphosulfate, Half-site, PAPS, sulfation, sulfotransferase, SULT, SULT1B1

## Abstract

The cytosolic sulfotransferases (SULTs) are dimeric enzymes that catalyze the transformation of hydrophobic drugs and hormones into hydrophilic sulfate esters thereby providing the body with an important pathway for regulating small molecule activity and excretion. While SULT dimerization is highly conserved, the necessity for the interaction has not been established. To perform its function, a SULT must efficiently bind the universal sulfate donor, 3′-phosphoadenosine-5′-phosphosulfate (PAPS), and release the byproduct, 3′, 5′-diphosphoadenosine (PAP), following catalysis. We hypothesize this efficient binding and release of PAPS/PAP may be connected to SULT dimerization. To allow for the visualization of dynamic protein interactions critical for addressing this hypothesis and to generate kinetically testable hypotheses, molecular dynamic simulations (MDS) of hSULT1B1 were performed with PAPS and PAP bound to each dimer subunit in various combinations. The results suggest the dimer subunits may possess the capability of communicating with one another in a manner dependent on the presence of the cofactor. PAP or PAPS binding to a single side of the dimer results in decreased backbone flexibility of both the bound and unbound subunits, implying the dimer subunits may not act independently. Further, binding of PAP to one subunit of the dimer and PAPS to the other caused increased flexibility in the subunit bound to the inactive cofactor (PAP). These results suggest SULT dimerization may be important in maintaining cofactor binding/release properties of SULTs and provide hypothetical explanations for SULT half-site reactivity and substrate inhibition, which can be analyzed in vitro.

## Introduction

The cytosolic sulfotransferases (SULTs) are important for the sulfate conjugation of an assortment of small molecules including hormones, pharmaceutical agents, and environmental toxicants, and therefore are classically regarded as Phase II drug-metabolizing enzymes (Evans and Relling 1999). Each active isoform catalyzes the transfer of a sulfuryl moiety from the obligate donor, 3′-phosphoadenosine 5′-phosphosulfate (PAPS), to an acceptor hydroxyl or primary amine moiety on the recipient substrate (Negishi et al. 2001). Addition of the sulfuryl to the hydrophobic substrate generally modifies its biological activity, increases the compound’s water solubility, and increases its excretion from the cell and body (Kotov et al. 1999; Cook et al. 2009).

Fourteen human SULT isoforms have been identified and the structures of twelve of these isoforms have been resolved to date (Otterness et al. 1992; Wilborn et al. 1993; Zhu et al. 1993; Aksoy et al. 1994; Wood et al. 1994; Her et al. 1996, 1997, 1998; Fujita et al. 1997; Sakakibara et al. 1998; Falany et al. 2000; Freimuth et al. 2004; Allali-Hassani et al. 2007). The architecture of each isoform is conserved with respect to primary, secondary, tertiary, and quaternary orders of structure, supporting kinetic reports of a conserved reaction mechanism (Allali-Hassani et al. 2007; Weitzner et al. 2009; Tibbs et al. 2014). Despite their structural conservation, the isoforms exhibit distinct substrate specificities that are difficult to define (Allali-Hassani et al. 2007). This complex substrate selectivity pattern is partially a result of the SULT’s sequential mechanism that requires the enzyme to form a ternary complex with both cosubstrates before catalysis (Leyh 1993). Loop 3, a region of the enzyme overlaying the active site, undergoes rearrangement upon the binding of PAPS, altering the shape of the enzyme’s active site (Cook et al. 2010b, 2012). The rationale for the conservation of this coordinated shift has not been investigated, but its effect on the binding and sulfation of particular substrates has been described (Cook et al. 2010b, 2013). Some substrates bind to the SULT in a manner independent of the presence of PAPS (noncooperativity), while other substrates display positive or negative cooperative binding with respect to PAPS (i.e., PAPS binding first either increases or decreases the productive binding rate of the substrate) (Sacco and James 2005; Allali-Hassani et al. 2007; Cook et al. 2012).

**Figure 1:**
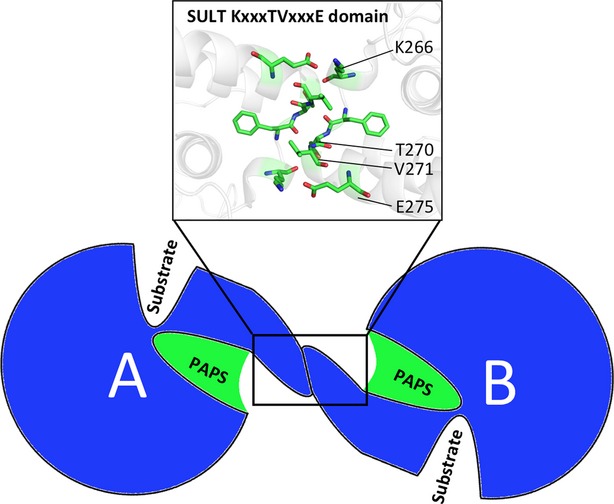
The relative locations of the dimerization domain and substrate-binding domains of SULTs. A cartoon illustration shows the PAPS-binding domain (green) of each dimer subunit (A and B) is near the dimer interface, with key residues KxxxTVxxxE forming antiparallel interactions.

Each human SULT isoform is a physiological homodimer whether or not it is bound to a ligand (Petrotchenko et al. 2001; Rehse et al. 2002; Weitzner et al. 2009). The two dimeric subunits interface in an antiparallel orientation along a small, highly conserved, motif near the C-terminus with the consensus amino acid sequence KxxxTVxxxE (Fig.1) (Petrotchenko et al. 2001; Pan et al. 2008; Weitzner et al. 2009). Despite the conservation of SULT dimerization, no suitable functional rationale for subunit oligomerization has been identified. The literature contains reports of monomeric SULTs (primarily generated via mutation) displaying altered substrate inhibition patterns and increased vulnerability to heat denaturation with minimal impact on sulfation activity (Lu et al. 2009; Cook et al. 2010a). Notably, three SULT isoforms (bovine SULT1A1, hSULT1E1, and hSULT2A1) have been described as exhibiting “half-site reactivity,” a phenomenon in which only half of the catalytic subunits catalyze the reaction at any given time (Beckmann et al. 2003; Sun and Leyh 2010; Wang et al. 2014). This phenomenon was first reported over 40 years ago and is a fairly common attribute of enzyme families including aldehyde dehydrogenases, thymidylate synthases, and biotin carboxylases (Matthews and Bernhard 1973; Anderson et al. 1999; Mochalkin et al. 2008; Yoval-Sanchez et al. 2013). To exhibit such a mechanism, the subunits of an oligomeric complex, like the SULT dimer, are required to “communicate” with one another in a coordinated fashion. The overall conservation and proximity of the dimerization domain to the PAPS-binding domain lend themselves to the hypothesis that the binding of PAPS, which is already known to elicit changes to SULT structure, may confer structural changes that are communicated to the neighboring subunit. A detailed description of this mechanism could provide an explanation for the preservation of human SULT dimerization as well as offer insight toward other SULT phenomena such as substrate inhibition.

To our knowledge, a structural study targeted toward describing the effects of PAPS binding on SULT dimers has not been reported. The visualization of protein dynamics and the effects of PAPS/PAP binding to a single dimer subunit are critical in addressing this hypothesis. Thus far, standard structural methods have fallen short in their ability to provide information on SULT dynamics and dimer asymmetry. To overcome the limitations of standard structural methods, molecular dynamic simulations (MDS) of hSULT1B1, a prototypical human SULT, were conducted in the presence and absence of PAPS or PAP to analyze the dynamic shifts induced by cofactor binding and evaluate their effects on the dimeric partner. The results of this study offer valuable insights into the complex catalytic mechanism of SULTs, which will be the subject of in vitro investigations in the immediate future.

## Materials and Methods

### Design

**Figure 2:**
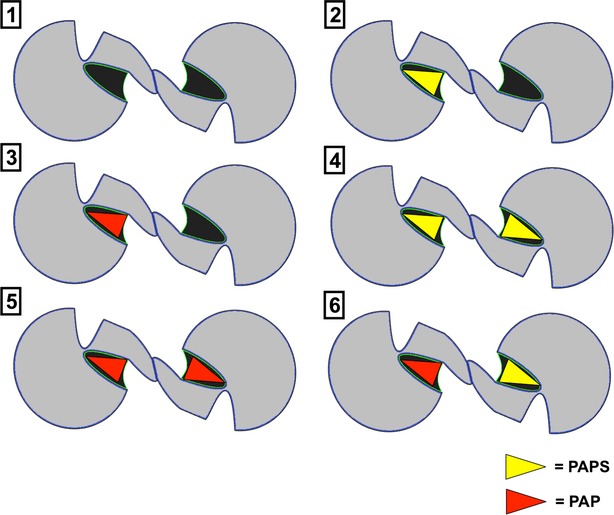
The six different PAP/PAPS-binding combinations displayed by SULT dimers. [1] Neither subunit bound to cofactor, [2] one subunit bound to PAPS, [3] one subunit bound to PAP, [4] both subunits bound to PAPS, [5] both subunits bound to PAP, [6] or one subunit bound to PAPS, and the other bound to PAP. The small molecules are depicted as yellow triangles (PAPS) or red triangles (PAP).

Each SULT dimer can bind PAPS or PAP in six different combinations. Therefore, six systems were prepared for MDS with each combination of PAP/PAPS bound to the hSULT1B1 dimer, depicted more precisely in Figure2. The number accompanying each system in Figure2 will be used to reference the system throughout this manuscript.

Human SULT1B1 crystal structure 3CKL, resolved in complex with PAP and resveratrol, served as the base model for this study (Pan et al. 2008). The Molecular Operating Environment (MOE) suite of programs and PYMOL were used for structure preparation (Schrodinger_LLC 2010; Chemical_Computing_Group_Inc 2013). Visual Molecular Dynamics 1.9.1 (VMD) served as the platform for the final preparation of the system for MDS as well as for trajectory analysis (Humphrey et al. 1996). CHARMM22 parameters were used for all polypeptides, while parameters for small molecules (PAP or PAPS) were calculated on the SwissParam server (Brooks et al. 2009; Zoete et al. 2011). MDS was performed with NAMD 2.7 (Not Another Molecular Dynamics (program)) on the Alabama Supercomputer (ASC) (Phillips et al. 2005). Unless stated otherwise, the default plugin/program values were retained.

### SULT1B1/PAPS model preparation

The hSULT1B1 structure (PDB: 3CKL) contains two polypeptide chains interacting along a noncanonical dimerization interface (Pan et al. 2008). This interface is most likely a product of crystal packing because multiple studies have described the dimerization domain as a conserved motif near the C-terminus with the sequence KxxxTVxxxE (Petrotchenko et al. 2001; Weitzner et al. 2009). The canonical dimerization interface was revealed upon generation of crystal cell mates using the PDB symmetry operations and confirmed on the PDBePISA interface server. The canonical interfacing subunits were retained as the relevant SULT1B1 dimer structure, and are referred to as subunits/chains “A” and “B” throughout this manuscript.

All ions, water molecules, and small molecules (PAP and resveratrol) were removed from the PDB file. Missing aa atoms were replaced and guided into place by energy minimization via the AMBER99 forcefield in MOE (Wang et al. 2004). The existing crystallographic atom coordinates were retained throughout energy minimization. The system was then protonated using the MOE Protonate 3D function at a pH of 7.4 (Chemical_Computing_Group_Inc 2013). This model was submitted to the NIHSAVes server for analysis by PROCHECK, WHAT_CHECK, ERRAT, VERIFY_3D, and PROVE to validate its quality (Luthy et al. 1992; Colovos and Yeates 1993; Laskowski et al. 1993; Hooft et al. 1996; Pontius et al. 1996). The final model received favorable scores (Verify_3D 100% >0.2, Errat quality factor 96.9)

The coordinates of the PAP atoms were retained from the original crystal structure and saved as an independent file. The MOE builder function was used to model a sulfate moiety onto the 5′ phosphate group of PAP to generate a relevant structure of PAPS within each dimer subunit. These PAP/PAPS molecules were then subjected to charge correction, protonation, and energy minimization within each respective protein pocket using the AMBER12:EHT forcefield (Wang et al. 2004; Case et al. 2005). Atomic parameters and structure files (PSFs) were then generated for each small molecule as described above.

### System preparation

All further preparation of the system for MDS was performed in VMD (Humphrey et al. 1996). First, a PSF was generated for each individual protein chain using the PSFGEN plugin and CHARMM22 topology definitions (Brooks et al. 2009). Explicit water (TIP3) was generated using the VMD solvate plugin; the boundaries of which were located 15 Å outside of the most distal portion of the protein in all directions to avoid protein self-interaction across the periodic boundaries. The partial charge of the system was then calculated and the system charge was annulled by addition of Na^+^ ions before addition of 150 mmol/L Na^+^ and Cl^−^ to recapitulate an in vivo environment.

### Molecular dynamics simulation

Prior to productive MDS, the system was energy minimized in a conjugate gradient manner. Thermal energy was added to the system, stepwise, for a total of 1.5 nsec until the temperature reached 300 K. Each system was then simulated at constant temperature and pressure (1 atm) for 50 nsec, using NAMD 2.7. Trajectories were downloaded and analyzed using VMD 1.9.1 (Humphrey et al. 1996).

### Analysis

The systems were aligned according to the root mean square deviation (RMSD) values of the protein *α*-carbon atoms. Each system was considered “equilibrated” when the slope of the protein RMSD versus time curve was essentially zero. Only frames following this point of equilibration were used for analysis. When necessary, the system was aligned according to the RMSD values of each individual subunit, such as in the case for the analysis of individual dimer subunit properties and measuring relative movement between subunits.

It is unreasonable to expect any randomly selected single frame from the simulation to confidently display the effects of PAPS/PAP-binding on the structure of the SULT dimer. Therefore, the average position of each atom over 10 nsec increments was calculated, and the resulting atom locations saved as independent PDB files. These “averaged” structures allowed for direct comparison and identification of large structural divergences identifiable by visual examination.

The dynamic flexibility of different regions of each subunit are of particular concern in this study as specific regions of the SULTs are known to undergo coordinated dynamic shifts in the presence of the cofactor, PAPS (Cook et al. 2010b). To quantify the flexibility/mobility of different regions of SULT1B1 in the presence and absence of PAP or PAPS, the root mean square fluctuations (RMSF) were calculated for the *α*-carbon atoms of the protein. For this type of calculation, a reference position for a particular residue was selected and the deviation from this reference position was calculated over time. Using this information, an overall flexibility factor was calculated for each individual subunit by averaging the total RMSF values from all residues excluding the ten N- and C-terminal residues. The movements of the terminal regions of the protein were highly unpredictable and could potentially skew/mask results, and therefore were excluded.

To assess the extent of structural flexibility as well as identify any structural alterations induced by PAPS or PAP binding, the changes in secondary structure over the entire trajectory were monitored. These values were recorded as the percentage of time each residue adopted a particular secondary structure (e.g., *α*-helix, extended *β*-sheet). As a baseline reference, the time each residue adopted each secondary structure was averaged for every simulation. Comparison of each individual simulation to this average allowed for the identification of structural divergences induced by PAP or PAPS binding.

## Results

### Root mean squared fluctuation

**Figure 3:**
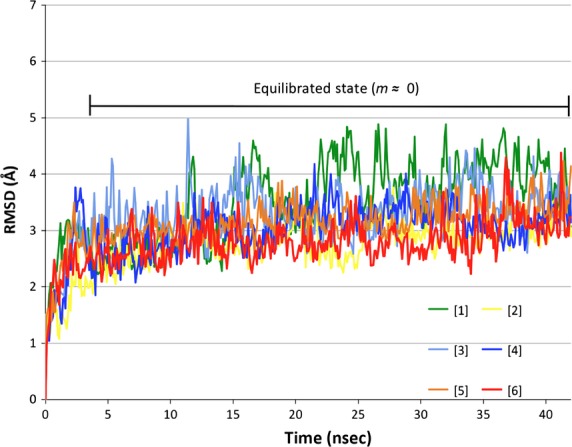
Root-mean-square deviation of protein *α*-carbon atoms over 43 nsec of simulation. Data were analyzed following system equilibration (slope (m) ≈ 0, indicated on graph). Green = simulation [1], yellow = simulation [2], light blue = simulation [3], royal blue = simulation [4], orange = simulation [5], red = simulation [6].

**Figure 4:**
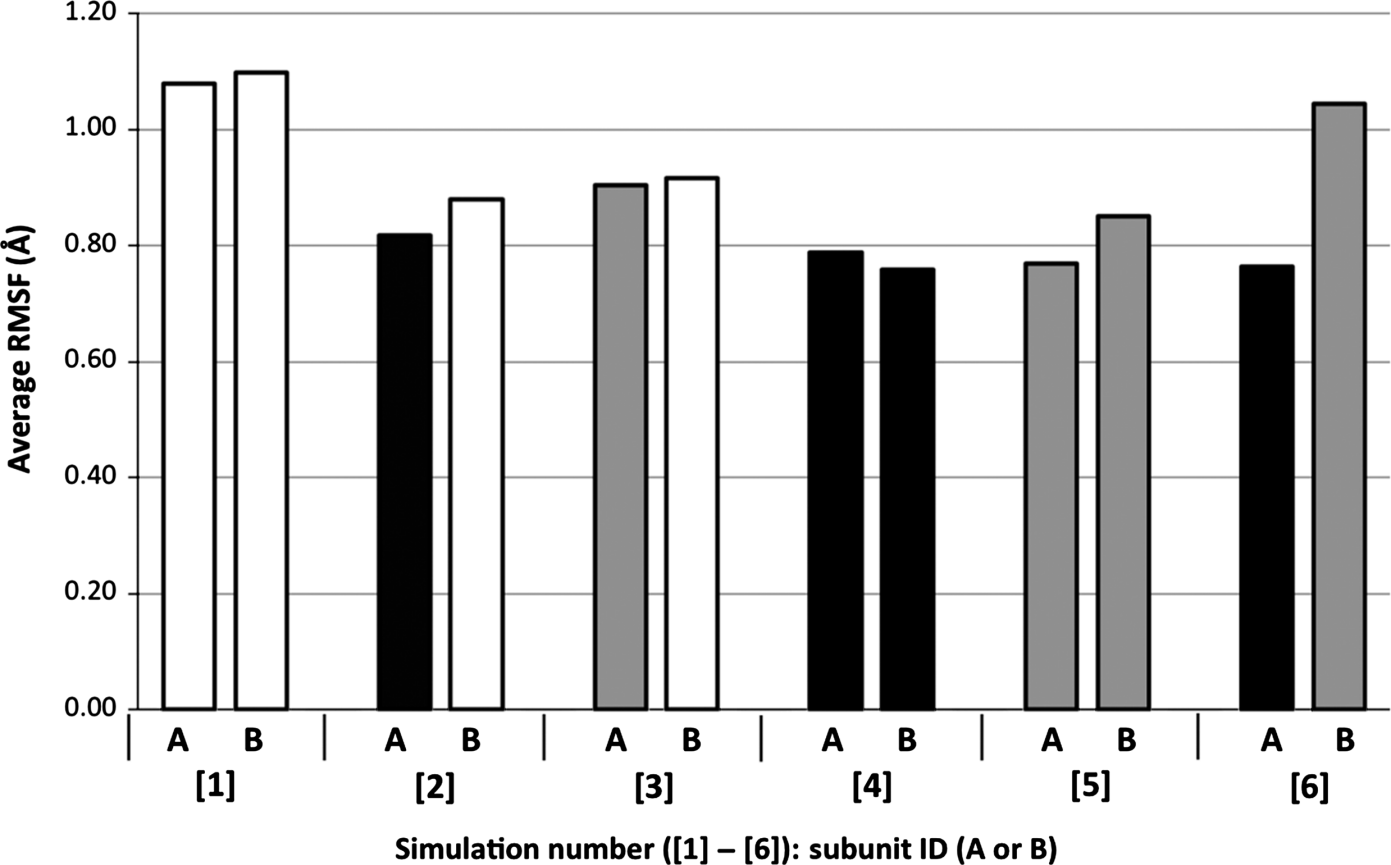
Average *α*-carbon RMSF values for both hSULT1B1 dimer subunits (A and B). The color of each bar represents binding state of the subunit: White = No cofactor, Black = PAPS, Grey = PAP.

Following equilibration (Fig.3), the RMSF of each *α*-carbon atom (excluding the ten N- and C-terminal residues) was calculated. Average RMSF values for each subunit are reported in Figure4, providing a quantitative description of the overall flexibility of each hSULT1B1 subunit. Each subunit of the fully apo dimer displayed the highest average flexibility with RMSF values of 1.08 and 1.1 Å (subunits A and B, respectively). Binding of PAPS or PAP to a single chain of the dimer decreases the flexibility in both the bound subunit and its unbound partner (Fig.4). PAPS (0.82 Å) appears to decrease the protein flexibility more than PAP (0.90 Å). Saturation of both dimeric subunits with either PAPS or PAP further decreases the flexibility of the enzyme (PAPS Avg. 0.78 Å, PAP Avg. 0.81 Å). When each subunit of the dimer is differentially bound to PAPS and PAP, the PAPS-bound chain is in the most stable state (0.76 Å), while the partnering PAP-bound chain is significantly more unstable (1.04 Å).

**Figure 5:**
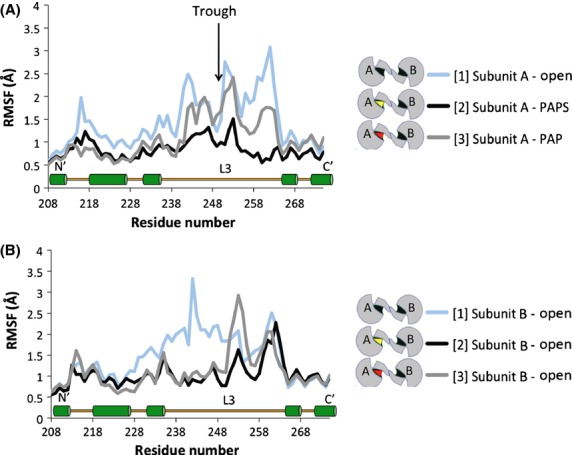
Effects of PAPS/PAP-binding on the mobility (RMSF) of amino acids 208–273, including Loop 3 (aa 235–268) and a portion of the dimerization domain (aa 266–275). (A) Subunit A of simulations [1], [2], and [3]. (B) Subunit B of simulations [1], [2], and [3]. A relatively high level of stability was observed for residue 250 across simulations (trough indicated by black arrow). The key indicates the PAP/PAPS-binding state of each subunit. A secondary structure map of the highlighted region is available toward the bottom of the graph (L3 = Loop 3, green cylinders = *α*-helix).

The change in the average RMSF values for each chain is most likely the result of a change in the dynamics of specific portions of the protein. Therefore, the relative mobility of each residue along the backbone of the protein was compared. Specific regions of the protein showed significant alterations in response to PAP or PAPS binding. One of these regions was Loop 3. Figure5 depicts the mobility of Loop 3 (aa 235–263) in simulations [1], [2], and [3]. Direct comparison of subunit A from each simulation shows the presence of either PAP or PAPS decreases the flexibility of Loop 3 in the vicinity of residue 262, directly adjacent to the KTVE dimerization domain. It exhibits an RMSF of 3.07 Å in the apo enzyme (simulation [1]), while the presence of PAPS or PAP reduces this RMSF value to 0.6 and 1.75 Å, respectively (simulations [2] and [3]). Regardless of the presence of the cofactor, relative flexibility, represented by a trough in the RMSF line, was observed at residue 250 (Asp) across all simulations (Fig.5A and B). This trough separates Loop 3 into two halves; the N-terminal half overlaying the substrate binding domain and the C-terminal half overlaying the PAPS binding domain. In subunit B in simulations [1], [2], and [3] (all of which are in an apo state), residue 262 exhibited similar mobility. The N-terminal half of Loop 3 is highly mobile in chain B of simulation [1], while it is stable in simulations [2] and [3].

**Figure 6:**
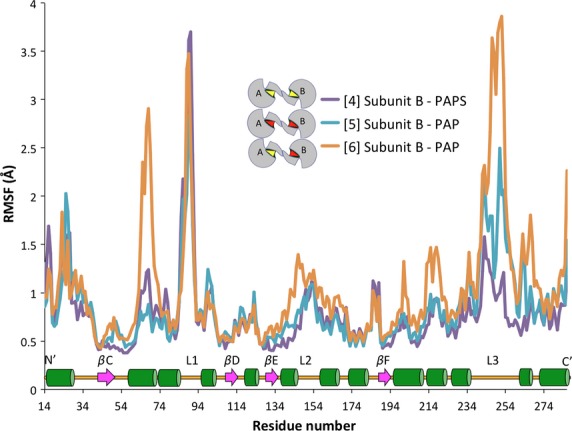
Comparison of the mobility (RMSF) of each subunit B residue in three simulations ([4], [5], and [6]). As illustrated in the key, orange and teal both indicate a chain bound with PAP – the partnering chain of each is bound with PAP and PAPS, respectively. Purple indicates a PAPS-bound chain partnered with another PAPS-bound chain. A secondary structure map of the highlighted region is available toward the bottom of the graph (green cylinders = *α*-helix, magenta arrows = *β*-sheet backbone, L1/2/3 = Loops 1/2/3).

Figure6 depicts the complete RMSF of subunit B when the dimeric enzyme is fully saturated with cofactor (either PAP or PAPS). This figure shows that Loop 3 is highly mobile (approximately 3.9 Å) for chain B in simulation [6] relative to simulations [4] and [5]. A second region, upstream of Loop 1 (aa 63-72), is also more mobile throughout this simulation relative to the control simulations. The increased mobility of these two regions is the largest contributor to the general flexibility of this chain depicted in Figure4. The outlier (chain B of simulation [6]) is PAP-bound but is paired with a partnering subunit that is PAPS-bound while both chains of hSULT1B1 are saturated with PAPS and PAP in simulations [4] and [5], respectively. The following analyses were designed to further understand the cause of the flexibility to these particular regions of hSULT1B1 to evaluate its potential involvement in the hSULT1B1 mechanism.

### Observational changes

**Figure 7:**
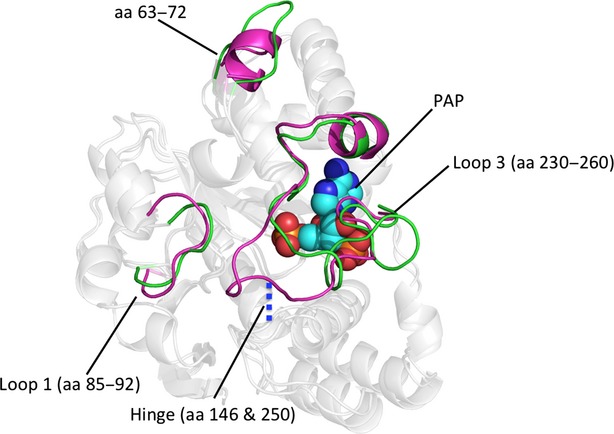
Observational shifts in hSULT1B1 structure. Loop 1 (aa 85-92), a region upstream of Loop 1 (aa 63-72), and Loop 3 show the most variability throughout the different simulations. PAP (teal spheres) is present for reference and orientation. A key *β*-bridge (blue dash) is lost in chain B of simulation [6] (green cartoon), but is present in chain B of simulation [5] (magenta cartoon) as well as all other simulations, resulting in high mobility of Loop 3.

Selection of individual frames for analysis could bias results, therefore, average protein structures over the last 10 nsec of simulation were compared. Figure7 shows the structural alterations that are primarily responsible for the observed RMSF alterations in chain B of simulation [6]. A critical *β*-bridge between Loop 3 and the base of the active site (Met146 and Asp250) is intact in chain B of simulations [4] (not shown) and [5], but appears to be nonexistent in chain B of simulation [6]. Residues 63-72 adopt an *α*-helical conformation in simulations [4] (not shown) and [5], but this region is unstructured in chain B of simulation [6] (Fig.7). Loop 1 adopts a similar conformation in all three simulations (Fig.7) and displays similar mobility in each simulation (Fig.6).

### Breaking the hinge interaction

**Figure 8:**
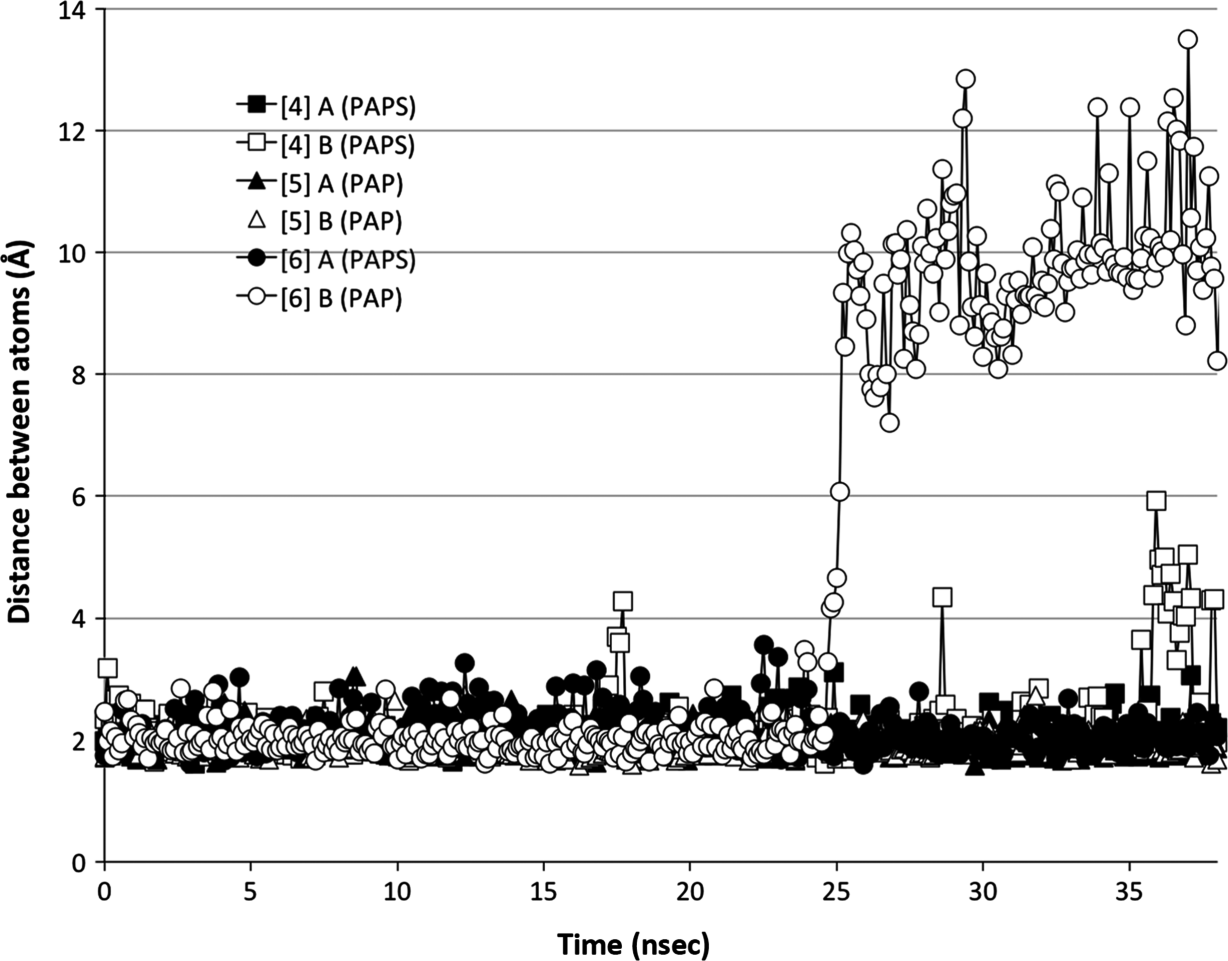
Broken hinge-region. Met146 and Asp250 form a hydrogen bond that ties Loop 3 to the base of the active site. This “hydrogen bond distance” was measured for chains A and B of simulations [4], [5], and [6] and plotted against the time frame at which it was measured. Simulation [4] is depicted as a square, simulation [5] as a triangle, and simulation [6] as a circle. Filled in shapes represent subunit A and hollow shapes represent subunit B.

Secondary structure analysis data (not shown) suggested that a key *β*-bridge between Met146 and Asp250 was intact throughout a majority of the simulations. To gauge the state of the hydrogen bond in simulations [4], [5], and [6], the distance between the relevant NH and CO bonding groups was measured throughout each trajectory. This distance is plotted against time in Figure8. In most simulations, the average distance was maintained just over 2 Å while at the 24.7 nsec time-point in chain B of simulation [6], this distance increased to 3.28 Å and continued to progress until the average distance was nearly 10 Å for the remainder of the simulation.

### PAP structure in the active site

**Figure 9:**
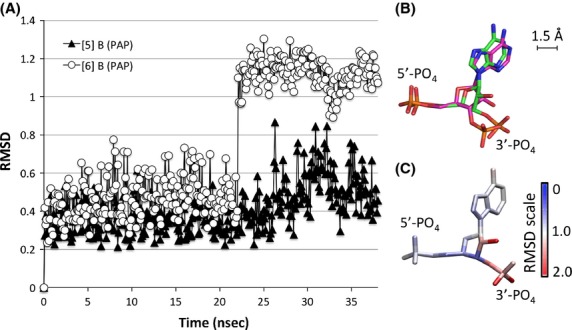
Change in PAP orientation within the hSULT1B1 active site. (A) The RMSD of PAP in simulation [6] (white circle) indicates time-point at which the structural shift occurred (22.1 nsec). The RMSD of PAP in Chain B of simulation [5] is included for reference (black triangle). (B) The average PAP orientation during the first 22.1 nsec is illustrated in green while the average PAP orientation for the remainder of the simulation is illustrated in magenta. (C) Each atom of the PAP structure is colored according to its RMSD value. Areas in red (e.g., the 3′ phosphate) display greatest divergence from the original conformation, while areas in blue remain unchanged.

Analysis of simulation [6] provided visual indication that PAP underwent a structural shift during the simulation. Therefore, the RMSD was calculated for the inactive cofactor. RMSD calculations show the shift occurs 22.1 nsec after the system reached equilibration (Fig.9).

The average orientation of PAP before and after this 22.1 nsec time-point was calculated. The position of certain areas of the molecule within the active site remained relatively unchanged, while others were shifted by nearly 2.5 Å (Fig.9). The RMSD was calculated between these two average structures to determine the portions of the molecule that underwent the greatest change. As seen in Figure9, the 3′ phosphate and nearby atoms displayed the greatest change, followed by the position of the adenosine group, while the position of the rest of the molecule was relatively unchanged.

**Figure 10:**
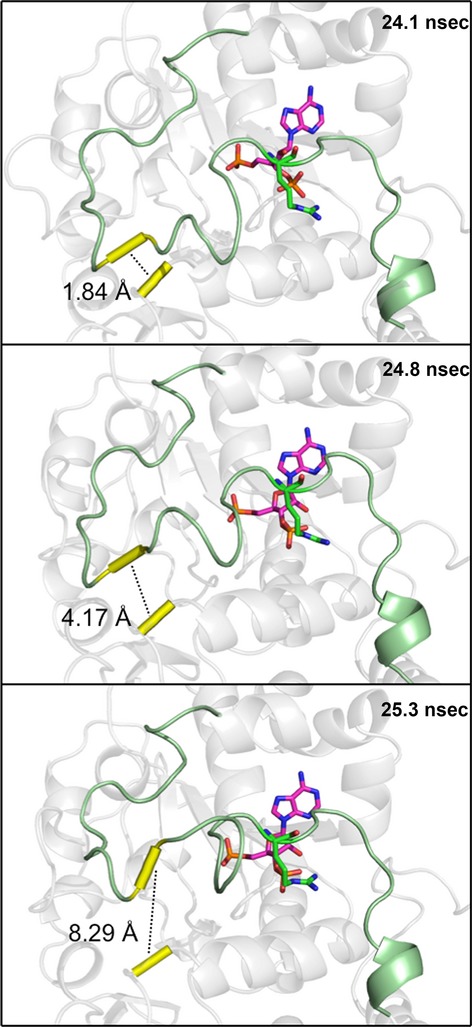
Visual indication of the broken hinge region. Shortly following a shift in the binding position of PAP (magenta sticks), a key hydrogen bond (dashed line) between Met146 and Asp250 (yellow tube) was broken, allowing Loop 3 (green) to be highly mobile. The shift in PAP appears to be translated to Loop 3 primarily by an interaction with a single residue (R258).

The shift in PAP orientation directly precedes the unhinging of Loop 3 (Fig.10). Loop 3’s interaction with the cofactor (PAP) is mediated by a single residue, R258, that is conserved across all SULTs. R258 directly interacts with the 3′-phosphate of PAP, the portion of PAP that underwent the largest shift in orientation. R258 maintains its interaction with the 3′-phosphate of PAP even after the molecule shifted (Fig.9).

## Discussion

SULT dimerization is a conserved phenomenon for which no suitable explanation has been identified. At least three SULTs act via a half-site reaction mechanism, implying dimeric SULT subunits communicate with each other through the KTVE domain (Beckmann et al. 2003; Sun and Leyh 2010; Wang et al. 2014). The proximity of the interface to the cofactor-binding domain suggests the binding of the cofactor may influence subunit interactions. Using MDS, the potential for hSULT1B1 dimeric subunits to communicate with one another was tested. The hSULT1B1 dimer subunits displayed the ability to do so in a manner dependent on PAP and PAPS binding, providing evidence that PAP(S) binding and release could drive subunit “cross-talk.”

The thermostability of at least eight human SULT isoforms is increased 8–10°C by the presence of PAP (Allali-Hassani et al. 2007). RMSF data show both hSULT1B1 dimer subunits are relatively unstable when neither chain is bound to the cofactor while the binding of PAP(S) to a single chain of the dimer decreases the flexibility of both subunits, even if the partnering subunit is in an unbound state (Fig.4). If the subunit acts fully independent of its partner, one would expect the binding of PAP(S) to one subunit to have no effect on its dimeric partner. RMSF data summarized in Figure4 show the subunits likely do not act with independence, but instead communicate through their only contact point, the KTVE domain, in a manner consistent with backbone flexibility.

Crystallographic reports have shown the binding of PAP(S) to SULTs stabilizes Loop 3 through direct interactions (Bidwell et al. 1999; Chang et al. 2004; Allali-Hassani et al. 2007; Lu et al. 2008). MDS of hSULT1B1 confirms the stabilizing effects of PAP(S) on Loop 3, located directly adjacent to the dimerization domain (Fig.5). Loop 3 is divided into two halves by a stable *β*-bridge interaction between Asp250 (Loop 3) and Met146 (bottom of the active site), a conserved interaction also reported for hSULT2A1 (Cook et al. 2013; Wang et al. 2014). The N-terminal section overlays the substrate binding domain and the C-terminal section overlays the PAPS binding domain, nearest the dimerization domain. The mobility of the C-terminal portion of the loop is directly dependent on the presence of PAP or PAPS (Fig.5A), mediated by R258’s direct interaction with the 3′ phosphate group. Alternatively, the N-terminal portion’s mobility may actually be dependent on the state of its dimeric partner. The N-terminal half of Loop 3 is less flexible when PAP or PAPS is bound to the subunit’s partnering chain, while the region is relatively unstable when partnered with a subunit that is not bound to PAP(S) (Fig.5B). Secondary structure analysis of hSULT1B1 revealed that K266 adopted a structured *α*-helix when PAPS or PAP was bound while it was unstructured in the absence of PAPS or PAP (data not shown). The direct interaction of K266 with E275 of the dimer partner, coupled with its responsiveness to cofactor binding, makes it a likely candidate for directly conveying information between subunits.

R258, a residue conserved across all human SULTs, not only conveys information regarding the presence or absence of PAP(S) within the enzyme pocket but also seems to provide the enzyme with a sensor for the orientation of PAP within the pocket. In simulation [6], PAP underwent a conformational shift midway through the simulation (Fig.9). The shift was communicated to the enzyme through R258’s direct interaction with the 3′ phosphate, the moiety of PAPS that underwent the largest shift (1.5 Å, 60^o^ rotation) (Fig.9). Within 2 nsec, the shift in R258’s position resulted in the loss of Loop 3’s *β*-bridge interaction with the bottom of the enzyme’s active site (Figs.8, 10). Loss of this *β*-bridge interaction and “unhinging” of Loop 3 caused the loop to be highly mobile, and was primarily responsible for the high average RMSF of this subunit compared to others that were bound to PAP(S) (Figs.4, 6). The precise cause of PAP’s structural shift is unknown, though this shift and unhinging of Loop 3 only occurred in a hSULT1B1 dimer subunit that was bound to PAP and partnered with a PAPS bound subunit. The shift in PAP orientation may have been a coordinated movement in response to enzyme structure, though it is possible the shift was spontaneous and the enzyme responded to the spontaneous shift in PAP’s position. If the shifts were coordinated, it is possible that the increased flexibility of the PAP-bound subunit provides a mechanism for hSULT1B1’s favorable release of the inactive cofactor, PAP. After all, hSULT2A1 exhibits a similar binding affinity for both PAP and PAPS, therefore, the enzyme may exhibit a mechanism to favor the binding of PAPS over the binding of PAP during its steady state (Gulcan and Duffel 2011; Wang et al. 2014).

**Figure 11:**
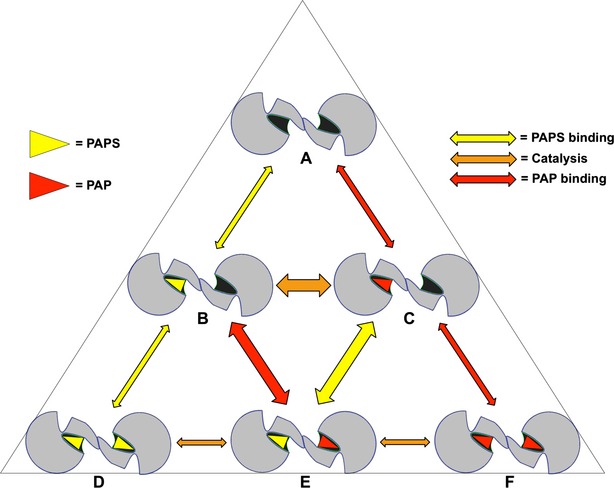
Transition of the dimeric SULT between cofactor binding-states. Yellow triangle = PAPS, red triangle = PAP, yellow arrow = PAPS binding/release, orange arrow = catalysis, red arrow = PAP binding/release.

To result in half-site reactivity, PAPS-induced cross talk must alter the PAPS-binding affinity or diminish the catalytic competency of the neighboring subunit. At least one report exists in which two distinct PAPS-binding affinities were observed for a dimeric rat sulfotransferase isoform, aryl sulfotransferase IV, suggesting PAPS binding to a single subunit induces structural changes in the dimeric subunit that alter the second site’s affinity for PAPS (Marshall et al. 1997). If only a single subunit is bound to PAPS, catalysis would be immediately followed by a state in which one dimer subunit is bound to PAP and the sulfated product. To the best of our knowledge, the effect of PAP’s presence in a single side of the dimer on the binding of PAPS to the second subunit has not been reported. After catalysis at the first site, the substrate and PAPS may bind with high affinity at the second site. After binding occurs at the second site, the first subunit may have to release PAP or “wait” for PAP to shift its orientation before the neighboring subunit is rendered catalytic. The shift in orientation of PAP in simulation [6] and unhinging of Loop 3 could be an integral part of this mechanism allowing the PAPS bound subunit to favor catalysis, while PAP release is favored in the dimer subunit. The favorable release of PAP, stimulated by PAPS’ presence in the neighboring subunit, could arm the SULT dimer with an oscillating mechanism as depicted in Figure11. When considering an in vitro environment where SULT and PAPS are incubated together prior to the addition of substrate, the dimer is free to bind either one or two molecules of PAPS depending on the concentration (Panel B or D). In either case, upon addition of substrate, a single subunit of the half-site reactive enzyme catalyzes sulfuryl transfer, forming PAP (Panel C or E). The enzyme is now free for PAPS to bind the neighboring subunit (Panel E). As illustrated in simulation [6], bound PAPS may stimulate an increase in Loop 3 flexibility in the PAP bound chain, favoring release of PAP, reestablishing a dimer complex with only one subunit bound to PAPS (panel B). This binding and release pattern can progress, oscillating between three distinct states, panels B, C, and E (Fig.11).

Further characterization of the oscillating mechanistic model could yield important insights in understanding SULT substrate inhibition (James 2014). The mechanism of substrate inhibition is a debated topic. Recent publications suggest involvement of a dead-end complex (enzyme:PAP:substrate) at high substrate concentrations, though this dead-end complex may be dependent on the reducing conditions under which the SULTs are often studied in vitro (Marshall et al. 2000; Gulcan and Duffel 2011; Wang et al. 2014). Participation of the SULT dimer in substrate inhibition has not been heavily considered. Considering a situation in which the substrate concentration is high (over the Km) and catalysis of the reaction is favorable, the model of cyclical binding and release of PAP(S) indicates the high substrate concentrations would push the equilibrium of the reaction toward catalysis prior to the release of PAP from the neighboring subunit. Quick catalysis (prior to the release of PAP from the neighboring subunit) would result in the formation of an “inactive” dimer with PAP bound to both subunits, negating PAPS docking (Fig.1F). Substrate may bind back to the enzyme to form an inactive complex and reaction progression would now be dependent on the passive off-rate of one of the PAP molecules from either side of the dimer. Uncoordinated release of PAP (F→C) “stalls” the enzyme resulting in a slower average reaction rate. This model of the involvement of SULT dimerization in substrate inhibition remains to be tested. However, it is worth noting that SULT isoforms have been reported to lose susceptibility to substrate inhibition upon monomerization (Cook et al. 2010a).

In this study, MDS provided a unique view of SULT dynamics in response to cofactor binding. While MDS provides a tool to overcome the limitations of crystallography, its own limitations cannot be ignored. The method is time/computationally-intensive, requiring relatively short simulation time frames. Further, chemistry (bond breaking/forming) cannot be considered in the simulations. Therefore, MDS could not be used to investigate the contribution of catalysis to the SULT mechanism. In spite of these limitations, MDS was well suited for the generation of testable hypotheses regarding the effects of PAP(S) binding on the SULT dimer. Dimeric hSULT1B1 subunits seemed to display the capability of communicating through the conserved dimerization domain in a manner dependent on cofactor binding thus providing a theoretical basis for SULT half-site reactivity. In conjunction with published data, results of this study allowed us to construct a model for this PAPS/PAP dependent communication involving SULT half-site reactivity. Importantly, this model is testable in vitro via a combination of careful binding and activity assays. If the oscillating mechanism described in this model is accurate, it could provide further insight into SULT phenomenon such as substrate inhibition and substrate selectivity.

## Abbreviations

Aa: amino acid
MDS: molecular dynamic simulation
PAP: 3′, 5′-diphosphoadenosine
PAPS: 3′-phosphoadenosine 5′-phosphosulfate
SULT: cytosolic sulfotransferase
